# Quantitative Classification of Uterine Myoma Perfusion on DCE-MRI: Retrospective Analysis of Data and Clinical Implications

**DOI:** 10.3390/diagnostics15121464

**Published:** 2025-06-09

**Authors:** Alan Bruszewski, Agnieszka Lach, Maciej Wilczak, Karolina Chmaj-Wierzchowska

**Affiliations:** Department of Maternal and Child Health and Minimally Invasive Surgery, Poznan University of Medical Sciences, 60-535 Poznan, Poland; lach.agnieszka95@gmail.com (A.L.); mwil@ump.edu.pl (M.W.)

**Keywords:** uterine myomas, dynamic contrast-enhanced magnetic resonance imaging (DCE-MRI), Funaki classification

## Abstract

**Background/Objectives**: The degree of vascularization of myomas plays an important role in both diagnosis and the selection of appropriate treatment. This is particularly relevant for minimally invasive therapies such as uterine artery embolization (UAE), high-intensity focused ultrasound (HIFU), or radiofrequency ablation (RFA) in uterine myomas, as their effectiveness is highest in well-vascularized lesions. This study aimed to analyze the perfusion of uterine myomas using dynamic contrast-enhanced magnetic resonance imaging and to develop a new quantitative classification of lesion vascularization, referencing the Funaki classification. **Methods**: The study included 56 female patients. Three parameters were determined for each lesion: the maximum signal enhancement (Ratio), time to peak, and mean signal intensity (Mean). A KMeans cluster analysis (*k* = 3) was performed, dividing the data into three groups corresponding to Funaki types I–III. **Results**: Significant differences were observed between the groups. Type III myomas were found only in older patients, which may be relevant when qualifying patients for vascularization-targeted therapies such as HIFU or radiofrequency ablation. **Conclusions**: The proposed classification may serve as a basis for automating the assessment of myomas and supporting clinical decision-making.

## 1. Introduction

Uterine myomas (leiomyoma uteri) are the most common benign neoplasms of the female reproductive organs. Their incidence in women of reproductive age is estimated at 20–40%, while autopsy studies have detected these lesions in up to 70–80% of postmenopausal women [1,2]. Although they are often asymptomatic, myomas can cause various clinical symptoms, including heavy menstrual bleeding, lower abdominal pain, a sensation of pressure on adjacent organs (such as the bladder or rectum), fertility issues, and recurrent miscarriages [3,4].

The degree of vascularization of myomas plays an important role in both diagnosis and the selection of the appropriate treatment. This is particularly relevant for minimally invasive therapies such as uterine artery embolization (UAE), high-intensity focused ultrasound (HIFU), or radiofrequency ablation (RFA) of uterine myomas, as their effectiveness is highest in well-vascularized lesions [5,6,7,8].

The classic classification of myoma perfusion, proposed by Funaki, is based on the subjective analysis of signal curves obtained from dynamic contrast-enhanced magnetic resonance imaging (DCE-MRI) sequences, distinguishing three types of vascularization [9]. Despite its widespread use, this classification has limitations—most notably, the absence of objective quantitative criteria and reliance on the radiologist’s interpretation.

DCE-MRI enables the precise evaluation of tissue blood flow, including that of uterine myomas. By acquiring quantitative data—such as maximum signal enhancement, time to peak (TTP), and mean signal intensity—it is possible to develop a more objective and reproducible classification of vascularization, which could also be applied in automated analysis systems [10].

The purpose of this study was to develop a new quantitative classification of uterine myoma perfusion based on parameters obtained through DCE-MRI and analyzed using unsupervised machine learning (KMeans) in relation to Funaki’s classification. The results may serve as a foundation for creating tools that support diagnosis and therapeutic decision-making in daily clinical practice.

## 2. Materials and Methods

A retrospective analysis was conducted on contrast-enhanced pelvic MRI scans of patients hospitalized for uterine myomas at the Department of Maternal and Child Health and Minimally Invasive Operative Gynecology, Heliodor Święcicki Gynecological and Obstetrical Clinical Hospital of Karol Marcinkowski Medical University in Poznań, between June 2024 and March 2025.

Fifty-six female patients (aged 32–79) diagnosed with symptomatic uterine myomas (based on a history of abnormal uterine bleeding, anemia, and/or pelvic pain) were included in the study to exclude atypical lesions and plan further treatment, particularly minimally invasive options.

The eligibility criteria for contrast-enhanced pelvic MRI before radiofrequency ablation (RFA) included the presence of up to three uterine myomas, classified as FIGO types 2–5 and measuring less than 10 cm, in cases where there were contraindications to surgical treatment with other techniques and/or the patient did not consent to such treatments and when drug therapy had proven ineffective in relieving symptoms. The eligibility criteria also included patients with up to three myomas (FIGO types 0–3) undergoing assisted reproductive treatment in whom the myomas were considered likely to reduce the chance of pregnancy and hysteroscopic myomectomy had been ineffective.

The eligibility criteria for pelvic MRI before laparoscopic treatment (laparoscopic myomectomy) included the presence of uterine myomas classified as FIGO types 6–8 when there were contraindications to other treatment techniques and/or the patient did not consent to surgical treatment with other methods and drug therapy had failed to relieve symptoms.

The exclusion criteria for contrast-enhanced MRI included the presence of reproductive organ cancer, cervical dysplasia, severe systemic disease, pregnancy, contrast allergy, or severe renal impairment. All patients gave informed consent for contrast-enhanced pelvic MRI.

The imaging was performed using Siemens Healthineers Magnetom Sola 1.5T, Erlangen, Germany with a dynamic t1_GRASP_VIBE_FS sequence. Gadolinium contrast (0.1 mmol/kg b.w.) was administered intravenously at a rate of 2 mL/s.

Image analysis was conducted using Siemens syngoMR XA51 software. For each lesion, the region of interest (ROI) was carefully selected to represent the most characteristic area of the myoma, excluding adjacent tissues and boundary zones (such as the uterine wall or neighboring nonfibrotic structures), to avoid the distortion of signal values by elements unrelated to the lesion itself.

Three parameters were determined for each lesion:

- Ratio (%)—maximum signal amplification after contrast,
- TTP (s)—time to peak,
- Mean—mean signal intensity during the test.

The KMeans clustering algorithm (*k* = 3) was used to divide the data into three groups. These were then interpreted according to the traditional Funaki classification, as follows:

- Type I: rapid and intense amplification, short TTP,
- Type II: moderate amplification and average time to peak,
- Type III: low amplification, long TTP.

## 3. Results

The application of the KMeans clustering algorithm (*k* = 3) enabled the automatic classification of the data into three groups, corresponding to the three vascularization types in the Funaki classification. Type I included 28 patients aged 32–51 years (mean age, 43.54 ± 5.62 years), Type II included 24 patients aged 33–53 years (mean age, 43.92 ± 5.34 years), and Type III included 4 patients aged 41–79 years (mean age, 54.75 ± 16.66 years). The distribution of patient age across Funaki classification types is presented in Table 1.

The study found no significant differences between Type I and Type II according to Funaki and the age of the patients. Table 2 shows the age of the subjects according to the classification of Type I and Type II according to Funaki.

It was noted that Type III myomas occurred only in older patients (mean age, 54.8 years), suggesting a potential relationship between age and reduced vascularization. This may indicate the age-related degeneration of myomas, which could influence their responsiveness to treatments such as HIFU, RFA, or embolization. Type III lesions, characterized by weaker vascularization, may be less responsive to these therapeutic methods, which are most effective for highly vascularized lesions. Due to the small sample size and reduced susceptibility to treatment with HIFU, RFA, or embolization, this group was excluded from further analysis.

In Type I, the average Ratio was 156.96 ± 20.94 (Me = 156.89), the average TTP was 127.04 ± 42.05 (Me = 133.76), and the average Mean was 538.98 ± 55.51 (Me = 538.35). In Type II, the average Ratio was 122.04 ± 25.57 (Me = 113.85), the average TTP was 260.31 ± 33.91 (Me = 276.99), and the average Mean was 452.77 ± 8.38 (Me = 451.75). In Type III, the average Ratio was 37.60 ± 44.98 (Me = 33.41), the average TTP was 218.24 ± 89.10 (Me = 250.74), and the average Mean was 235.84 ± 111.76 (Me = 252.89). The mean values of perfusion parameters for each Funaki classification type are shown in Table 3.

In this study, a decrease in both Ratio and Mean values was observed from Type I to Type III, indicating a gradual decline in vascularization intensity. This trend reflects a progression from highly vascularized, biologically active myomas (Type I) to poorly vascularized lesions (Type III). The TTP value increased from Type I to Type II, which may suggest slower contrast enhancement dynamics in less vascularized myomas. In contrast, Type III exhibited a lower TTP value despite its low overall enhancement, possibly due to a limited dynamic enhancement range and the absence of a well-defined signal peak.

This approach provides a more objective and reproducible method for assessing myoma vascularization and supports the automation of diagnostic processes, contributing to the standardization of imaging evaluation. In this study, Type I myomas were found to have significantly higher Ratio values. Table 4 presents the Ratio measurements (%) for Type I and Type II lesions according to the Funaki classification.

Additionally, Type I myomas were characterized by significantly lower TTP values. Table 5 shows the TTP (s) measurements for Type I and Type II.

Type I myomas also exhibited significantly higher Mean signal intensity values. Table 6 shows the Mean measurements for Type I and Type II myomas according to Funaki.

**Figure 1:**
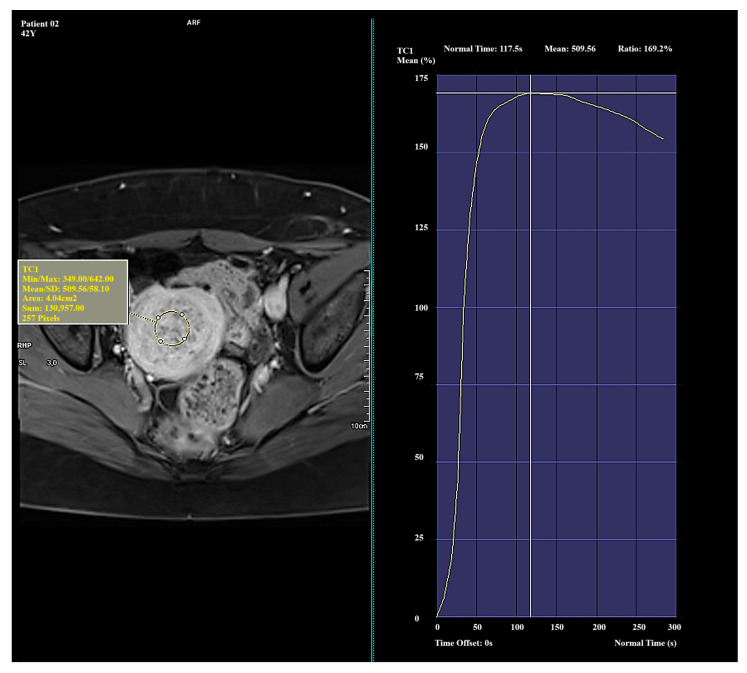
Example of amplification curve for Funaki Type I.

**Figure 2:**
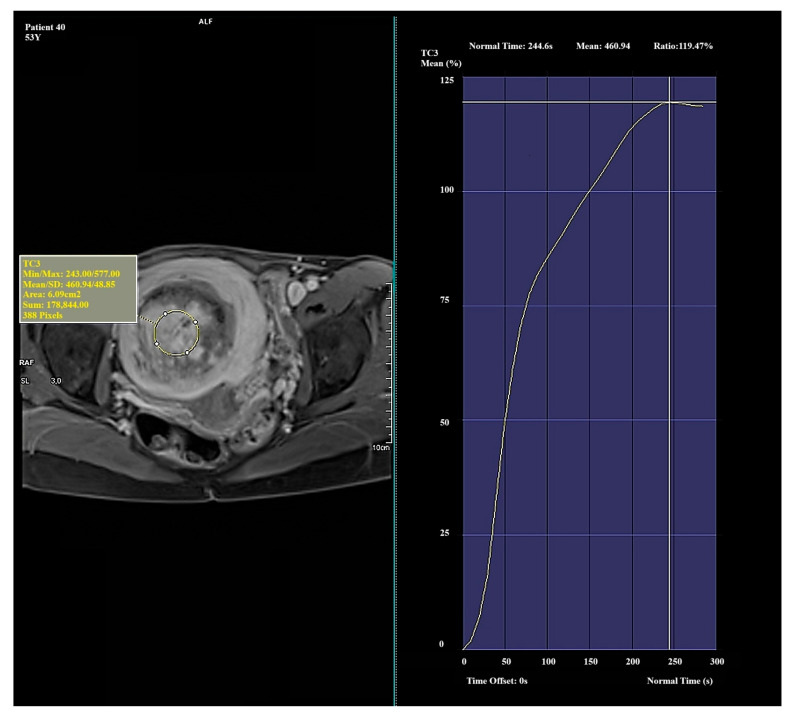
Example of amplification curve for Funaki Type II.

**Figure 3:**
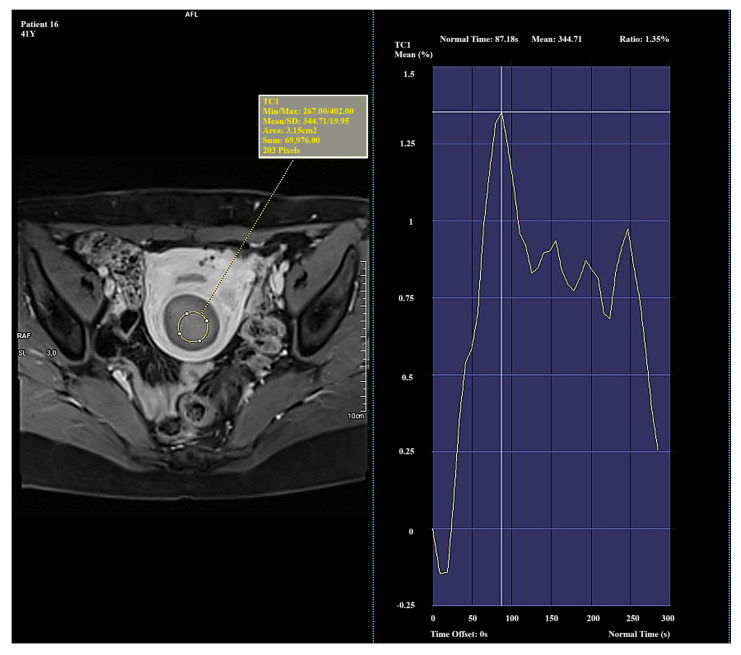
Example of amplification curve for Funaki Type III.

Significant and distinct differences in perfusion parameters were observed across the types. Type I lesions demonstrated strong and rapid enhancement and are likely the most biologically active. Type II lesions represented an intermediate category, showing clinical variability. Type III lesions—though rare in this study—displayed very low enhancement and signal intensity, which may correspond to fibrotic or ischemic changes. Due to the small number of Type III cases (*n* = 4), observations related to this group should be interpreted with caution and validated in a larger cohort. Representative enhancement curves for each Funaki classification type are illustrated in Figure 1, Figure 2 and Figure 3.

**Figure 4:**
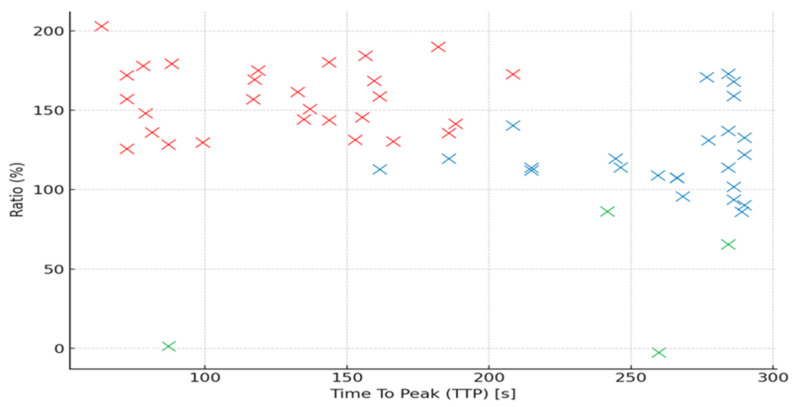
Scatterplot showing the distribution of perfusion parameters (Ratio vs. TTP) by Funaki type (red x—Funaki type I, blue x—Funaki type II, green x—Funaki type III).

The distribution of perfusion parameters (Ratio vs. TTP) by Funaki type is illustrated in Figure 4.

## 4. Discussion

This study demonstrates that the quantitative analysis of DCE-MRI parameters—such as Ratio, TTP, and Mean—can effectively classify uterine myomas in a manner consistent with the subjective Funaki classification. Rather than relying on the visual interpretation of enhancement curves, the analysis of these three parameters, which can be automatically extracted from Siemens syngoMR XA51 software, proves sufficient. These results represent a step toward objectifying the assessment of myoma perfusion and may have practical applications in planning noninvasive treatments.

Funaki et al. proposed a classification of myomas based on the shape of contrast enhancement curves observed in DCE-MRI—distinguishing Types I, II, and III according to signal intensity and enhancement dynamics [11]. In the present study, KMeans cluster analysis performed in three-dimensional space (Ratio, TTP, Mean) identified three groups that align with this classification. Type I myomas were characterized by a short TTP and high Ratio, Type II by moderate values for both parameters, and Type III by a low Ratio and delayed intensity peak. These findings confirm that the Funaki classification is reflected in objectively measurable, quantitative parameters.

The quantitative classification of uterine myoma perfusion holds significant clinical relevance, especially when considering noninvasive treatment methods such as high-intensity focused ultrasound (HIFU) or uterine artery embolization (UAE). Numerous studies have shown that the effectiveness of HIFU is highest in well-vascularized myomas, corresponding to Type I [12,13]. Higher perfusion allows for more efficient ultrasound energy dissipation and more effective tissue ablation. Similarly, in UAE, greater perfusion facilitates the selective occlusion of tumor vessels, thereby improving procedural outcomes [14,15].

In the present analysis, myomas with low perfusion (Type III) were more commonly observed in postmenopausal women, supporting the known relationship between hormonal activity and lesion vascularization [16]. These lesions often exhibit fibrotic and degenerative features [17], which may reduce the effectiveness of conservative treatment.

Previous quantitative studies, such as the work by Kim et al. [18,19], focused primarily on semiquantitative perfusion MRI parameters of uterine fibroids, including peak enhancement and wash-in slope, to evaluate treatment efficacy of MR-guided high-intensity focused ultrasound (HIFU) ablation. However, these studies did not apply classification methods to distinguish fibroid types, instead analyzing individual perfusion parameters in relation to therapeutic outcomes [18,19]. In contrast with these approaches, our method employs multivariate analysis and considers the full lesion volume, thereby enhancing representativeness and potentially improving classification accuracy.

This study has several limitations. First, the group corresponding to Type III myomas was small, limiting the statistical strength of the conclusions drawn for this subgroup. Second, the ROIs were manually delineated, which introduces subjectivity and interobserver variability. Future studies should consider implementing automatic segmentation techniques, such as U-Net-based neural networks. Third, the absence of histopathological confirmation for most lesions restricts the ability to directly correlate perfusion parameters with microscopic features.

Nevertheless, the findings may form the basis for developing tools to support clinical decision-making in selecting appropriate therapies for uterine myomas. Future research could include additional imaging parameters from other sequences (e.g., ADC values from DWI) and incorporate deep learning models to predict treatment response. The integration of these tools into hospital information systems (PACS, RIS) could enhance workflow for radiologists and interventional gynecologists.

The results indicate that Funaki’s classification can be effectively replicated using quantitative data derived from dynamic MRI. Moreover, the application of cluster analysis removes the element of subjective interpretation, making the classification more objective, reproducible, and potentially automatable.

This study confirmed that the quantitative analysis of perfusion parameters of uterine myomas obtained from DCE-MRI allows the objective classification of the vascularization of these lesions. Parameters such as maximum signal enhancement (Ratio), TTP, and mean signal intensity (Mean) provide a solid basis for the development of a new classification that eliminates the subjective interpretive elements inherent in the traditional Funaki classification. The developed classification can provide a starting point for standardizing the perfusion evaluation of myomas on MR imaging. By using unambiguous quantitative parameters, it will be possible to introduce harmonized diagnostic protocols, which can significantly improve the quality of imaging in daily clinical practice.

## 5. Conclusions

The quantitative analysis of perfusion parameters in DCE-MRI allows the effective classification of the vascularization of uterine myomas. Using the KMeans cluster algorithm, it was possible to distinguish three groups of lesions corresponding to Funaki’s classification, eliminating the subjective nature of traditional assessment. Parameters such as maximum signal enhancement (Ratio), TTP, and mean signal intensity (Mean) showed clear differences between types, confirming their diagnostic value.

The new quantitative classification not only allows for a more objective evaluation of lesions but also creates the possibility of automating the diagnostic process, which can significantly improve the quality and reproducibility of imaging in daily clinical practice. The results pave the way for the standardization of the assessment of myoma perfusion and further studies on the correlation with treatment response and histopathological results.

## Figures and Tables

**Table 1:** Mean age of patients with each Funaki type.

Funaki Type	Group Size	Mean Age
I	*n* = 28	43.54 ± 5.62 years (range 32–51)
II	*n* = 24	43.92 ± 5.34 years (range 33–53)
III	*n* = 4	54.75 ± 6.66 years (range 41–79)

**Table 2:** Occurrence of Type I and II according to Funaki classification vs. age of patients.

Age	*N*	*M* ± SD	Min–Max	Me [*Q*1–*Q*3]	U	*p*
I	28	43.54 ± 5.53	32–51	45 [40.5–48]	335.5	1
II	24	43.92 ± 5.33	33–53	43.5 [40.5–48]		
In total	52	43.71 ± 5.39	32–53	44 [40.5–48]		

**Table 3:** Average values of perfusion parameters in each Funaki type.

Funaki Type	Ratio (%)	TTP (s)	Mean
I	156.96 ± 20.94	127.04 ± 42.05	538.98 ± 55.51
II	122.04 ± 25.57	260.31 ± 33.91	452.77 ± 8.38
III	37.60 ± 44.98	218.24 ± 89.10	235.84 ± 111.76

**Table 4:** Type I and II according to Funaki vs. Ratio (%).

Ratio (%)	*N*	*M* ± SD	Min–Max	Me [*Q*1–*Q*3]	U	*p*
I	28	156.96 ± 21.21	125.62–202.82	156.9 [138.67–173.78]	96	<0.001
II	24	122.04 ± 25.02	85.99–172.84	113.85 [107.38–134.77]		
In total	52	140.84 ± 28.8	85.99–202.82	138.6 [116.67–168.18]		

**Table 5:** Type I and II according to Funaki vs. TTP (s).

TTP (s)	*N*	*M* ± SD	Min–Max	Me [*Q*1–*Q*3]	U	*p*
I	28	127.04 ± 41.98	63.63–208.47	133.76 [84.31–158.07]	8	<0.001
II	24	260.3 ± 36.76	161.55–289.95	276.99 [245.49–286.19]		
In total	52	188.55 ± 77.73	63.63–289.95	184.02 [125.72–272.47]		

**Table 6:** Type I and II according to Funaki vs. Mean.

Mean	*N*	*M* ± SD	Min–Max	Me [*Q*1–*Q*3]	t	*p*
I	28	538.98 ± 55.35	451.58–677.76	538.35 [497.04–580.08]	5.98	<0.001
II	24	452.77 ± 47.32	373.82–568.82	451.75 [425.25–476.13]		
In total	52	499.19 ± 67.19	373.82–677.76	493.66 [453.23–549.44]		

## Data Availability

The original contributions presented in this study are included in the article. Further inquiries can be directed to the corresponding author.
